# Nondisordered Cannabis Use Among US Adolescents

**DOI:** 10.1001/jamanetworkopen.2023.11294

**Published:** 2023-05-03

**Authors:** Ryan S. Sultan, Alexander W. Zhang, Mark Olfson, Muhire H. Kwizera, Frances R. Levin

**Affiliations:** 1 Department of Psychiatry, College of Physicians and Surgeons, Columbia University, New York, New York; 2 New York State Psychiatric Institute, New York; 3 Integrative Psychiatry, New York, New York; 4 Department of Epidemiology, Mailman School of Public Health, Columbia University, New York, New York

## Abstract

**Importance:**

Cannabis use is increasingly viewed by adolescents as not harmful. Youths with cannabis use disorder (CUD) are recognized by clinicians as being at risk for adverse outcomes, yet little is known about the associations between subclinical cannabis use (ie, nondisordered cannabis use [NDCU]) and adverse psychosocial events.

**Objective:**

To describe the prevalence and demographics of NDCU and to compare associations of cannabis use with adverse psychosocial events among adolescents with no cannabis use, NDCU, and CUD.

**Design, Setting, and Participants:**

This cross-sectional study used a nationally representative sample derived from the 2015 to 2019 National Survey on Drug Use and Health. Participants were adolescents aged 12 to 17 years, separated into 3 distinct groups: nonuse (no recent cannabis use), NDCU (recent cannabis use below diagnostic threshold), and CUD. Analysis was conducted from January to May 2022.

**Exposures:**

CUD, NDCU, or cannabis nonuse. NDCU was defined as endorsing recent cannabis use but not meeting the *Diagnostic and Statistical Manual of Mental Disorders *(*Fifth Edition*) (*DSM-5*) CUD criteria. CUD was defined using *DSM-5* criteria.

**Main Outcomes and Measures:**

The main outcomes were prevalence of adolescents meeting criteria for NDCU and associations between adverse psychosocial events and NDCU, adjusted for sociodemographic characteristics.

**Results:**

The 68 263 respondents (mean [SD] age, 14.5 [1.7] years; 34 773 [50.9%] males) included in the analysis represented an estimated yearly mean of 25 million US adolescents during 2015 to 2019. Among respondents, 1675 adolescents (2.5%) had CUD, 6971 adolescents (10.2%) had NDCU, and 59 617 adolescents (87.3%) reported nonuse. Compared with nonusers, individuals with NDCU had approximately 2 to 4 times greater odds of all adverse psychosocial events examined, including major depression (adjusted odds ratio [aOR], 1.86; 95% CI, 1.67-2.08), suicidal ideation (aOR, 2.08; 95% CI, 1.88-2.29), slower thoughts (aOR, 1.76; 95% CI, 1.58-1.96), difficulty concentrating (aOR, 1.81; 95% CI, 1.65-2.00), truancy (aOR, 1.90; 95% CI, 1.67-2.16), low grade point average (aOR, 1.80; 95% CI, 1.62-2.00), arrest (aOR, 4.15; 95% CI, 3.17-5.43), fighting (aOR, 2.04; 95% CI, 1.80-2.31), and aggression (aOR, 2.16; 95% CI, 1.79-2.62). Prevalence of adverse psychosocial events was greatest for adolescents with CUD (range, 12.6% to 41.9%), followed by NDCU (range, 5.2% to 30.4%), then nonuse (range, 0.8% to 17.3%).

**Conclusions and Relevance:**

In this cross-sectional study of US adolescents, past-year NDCU was approximately 4 times as prevalent as past-year CUD. A stepwise gradient association was observed for odds of adverse psychosocial events between adolescent NDCU and CUD. In the context of US normalization of cannabis use, prospective research into NDCU is necessary.

## Key Points

**Question:**

Is nondisordered cannabis use (NDCU) among US adolescents associated with adverse psychosocial events?

**Findings:**

In this cross-sectional study of 68 263 adolescents, NDCU was approximately 4 times more common than cannabis use disorder (CUD). NDCU and CUD were both significantly associated with adverse psychosocial events in a stepwise gradient manner.

**Meaning:**

These findings suggest that adolescents with NDCU or CUD had increased odds of adverse psychosocial events.

## Teaser

This cross-sectional study assesses the prevalence of nondisordered cannabis use among adolescents and associations of varying degrees of cannabis use with adverse psychosocial events among adolescents in the US.

## Introduction

Adolescence is a critical phase for brain development, and the endocannabinoid system plays an important role in fundamental brain development processes.^1^ Understanding how exogenous cannabis use may affect adolescents is critical, since cannabis use is the most widely used illicit substance among US adolescents and the primary reason for substance use treatment in this age group.^2^

Cannabis use is associated with deficits in cognitive and executive functioning, including processing speed, sustained attention, working memory, judgment and planning, problem-solving, decision-making, and self-regulation.^3,4,5,6^ Adverse mental health outcomes, including increased rates of depression and suicidal behaviors, have also been associated with youth cannabis use.^7,8,9^ Well-controlled longitudinal epidemiologic studies have demonstrated that adolescent cannabis exposure is associated with a 4-fold increase in adult psychosis diagnoses.^10^ Finally, cannabis use among youth is associated with worse academic performance and delinquent behavior.^11,12^

Past studies on adverse psychosocial associations among youth cannabis users have not differentiated risks between disordered cannabis use (those meeting criteria for *Diagnostic and Statistical Manual of Mental Disorders* [*Fifth Edition*] [*DSM-5*]^13^ cannabis use disorder [CUD]) and nondisordered cannabis use (NDCU; those below threshold criteria for a CUD diagnosis).^9,12,14,15,16,17^ As a result, little is known about the comparative strength of associations of disorder- and nondisorder-level cannabis use with adverse psychological events among young people.

Most US adolescents do not perceive regular cannabis use as harmful.^2,18^ Over the past decade, the perceived risk of harm of weekly cannabis use decreased by nearly half, from 47.5% to 27.4%.^2,19^ During the same period, cannabis use among individuals aged 12 years and older has increased from 11.6% to 17.9%.^19^ This trend is likely to continue, since decreases in negative perceptions of a substance are associated with increased use among adolescents.^20^ Finally, the recent shift in federal criminal justice policy to decriminalize marijuana possession may contribute to these trends.

Both the neuroscience and epidemiological characteristics of adolescent cannabis use support the clinical and public health importance of probing the associations of NDCU with adverse psychosocial events among US adolescents. If, for example, adolescents with NDCU have substantial likelihood of adverse psychosocial events, these findings would have implications for clinical assessment of cannabis use and classification of disordered cannabis use in adolescents.

To test this hypothesis, we assessed associations of NDCU and CUD compared with nonuse among adolescents across a range of adverse psychosocial events. The analysis focused on a nationally representative sample of adolescents (ages 12-17 years) from the 2015 to 2019 National Surveys on Drug Use and Health (NSDUH). Prior to conducting these analyses, we hypothesized that there could be a significant association between NDCU and adverse psychosocial events but to a degree lesser than that of CUD.

## Methods

### Study Population

This cross-sectional study analyzed responses from adolescents aged 12 to 17 years from the 2015 to 2019 NSDUH. Collected annually by the Substance Abuse and Mental Health Services Administration (SAMHSA), with approval by the institutional review board at Research Triangle Institute International, NSDUH is a cross-sectional national survey of the US population ages 12 years and older living in households and noninstitutional group quarters. All participants provided verbal informed consent. The classification of race and ethnicity in the NSDUH survey was based on the self-identification of respondents, using the racial and ethnic classifications established by the US Census Bureau, including African American, Asian, American Indian or Alaska Native, Hispanic, multiple races, Native Hawaiian or other Pacific Islander, White, and other.^2^ Race and ethnicity were included in analyses because they may confound the associations between cannabis use and psychosocial events. To select a nationally representative sample, SAMHSA used a multistage area probability sampling method. Additional details regarding NSDUH methods are available elsewhere.^21^ We evaluated the following sociodemographic factors: sex, race and ethnicity, grade level, community type, and total family income. We were unable to evaluate sexual orientation and gender identity, as they were not recorded for adolescents. Finally, the data set includes information on substance use disorders and major depression, but not other psychiatric disorders. This study is reported following the Strengthening the Reporting of Observational Studies in Epidemiology (STROBE) reporting guideline for cross-sectional studies.

### Measures

#### Degrees of Cannabis Use

Cannabis use was classified into 3 mutually exclusive categories, including nonuse, NDCU, and CUD. Cannabis use descriptions and codebook variables are included in eTable 1 in Supplement 1. Respondents who denied cannabis use ever or endorsed cannabis use more than 12 months ago were classified as nonusers. Respondents who endorsed cannabis use either within the past 30 days or between 30 and 365 days ago and did not meet CUD criteria according to the *DSM-5* were classified as having NDCU. Respondents who met *DSM-5* past-year use disorder criteria for cannabis^13^ were classified as having CUD. The NSDUH relies on *DSM-5*^13^ criteria to define CUD.

#### Adverse Psychosocial Events

We identified 9 potential adverse psychosocial events among adolescent cannabis users: major depressive episode (MDE), suicidal ideation (SI), slowed thinking, difficulty concentrating, truancy, low grade point average (GPA), being arrested, serious fighting, and physical aggression. Criteria descriptions and codebook variables for adverse psychosocial events are included in eTable 2 in Supplement 1. These measures were selected based on substantial past literature supporting associations with adolescent cannabis use.^9,12,14,15,16,17,22,23,24^

#### Poor Mental Health

MDE was defined as meeting *DSM-5* criteria for MDE^13^ within the past year. SI was defined as having had thoughts of ending one’s life during a recent period.

#### Cognitive Deficits

Cognitive measures of slower thoughts and difficulty concentrating were examined. Slower thoughts was defined as endorsing recent slowed or impaired thinking, and difficulty concentrating, endorsing recent trouble focusing on present tasks.

#### Low Academic Performance

Two measures of academic performance were evaluated. First, truancy was defined as adolescents who skipped 1 or more days of school in the past month. Second, low GPA was defined as adolescents with past semester GPA of C+ or below (ie, <80%), which has face validity as a natural cutoff.

#### Delinquent Behavior

Three delinquency measures were examined. First, breaking the law, defined as at least 1 instance of being arrested and charged for illegal activity in the past year. Second, serious fighting, having been in 1 or more serious fights in the past year. Third, physical aggression, at least 1 instance of an attack with intent to harm in the past year.

### CUD Criteria Analysis

To compare differences between NDCU and CUD according to severity as classified by the *DSM-5*,^13^ we first obtained 10 individual *DSM-5* symptom items for CUD, consistent across 2015 to 2019 NSDUH. We then obtained the mean number of symptom items met by adolescents in each group.

### Statistical Analysis

First, the adolescent sample was partitioned into mutually exclusive cannabis use categories: nonuse, NDCU, and CUD. Next, we computed unadjusted odds ratios (ORs) in logistic regression for NDCU and CUD use groups, using the nonuse group as the reference group. This process was repeated for each adverse psychosocial measure. To provide values representative of the US adolescent population, we incorporated NSDUH individual respondent sampling weights.^21^ Sampling weights were used in obtaining demographic prevalence values and in all subsequent analyses. Adjusted ORs (aORs) were obtained using the same methods while controlling for age, sex, race and ethnicity, and alcohol use disorder (AUD). Unadjusted OR values were included to inform clinician’s assessments of adverse psychosocial events in the community. aOR values were included to define independent associations of degree of cannabis use with psychosocial measures.

Statistical differences in strength of association with adverse events between NDCU and CUD groups were determined by nonoverlapping 95% CIs. Significant difference between NDCU and CUD vs nonuse was determined by 2-tailed *P* < .05. All logistic regression models were created in R statistical software version 3.5.1 (R Project for Statistical Computing) using the svydesign function from the survey package to generate ORs, aORs, and 95% CIs. Data were analyzed from January to May 2022.

## Results

### Sociodemographic Characteristics and Distribution of Cannabis Use Groups

From a total sample of 68 263 respondents (mean [SD] age, 14.5 [1.7] years; 34 773 [50.9%] males), representing an estimated yearly mean of 25 million US adolescents during 2015 to 2019, we classified individuals by cannabis use. Most respondents (59 617 respondents [87.3%]) denied any or recent cannabis use; 6971 respondents (10.2%) endorsed NDCU, and 1675 respondents (2.5%) met criteria for CUD (Table 1).

**Table 1.** Sociodemographic Characteristics of US 2015 to 2019 NSDUH Adolescent Respondents, by Type of Cannabis Use^a^

Characteristic	Respondents, No. (%)			
	Total (N = 68 263)	Classification of cannabis use		
		Nonuse (n = 59 617)^b^	Nondisordered cannabis use (n = 6971)^c^	Cannabis use disorder (n = 1675)^d^
2015-2019 mean national prevalence	24 906 678 (100)	21 752 144 (87.3)	2 543 257 (10.2)	611 277 (2.5)
Sex				
Male	34 773 (50.9)	30 446 (51.1)	3440 (49.4)	882 (52.6)
Female	33 490 (49.1)	29 171 (48.9)	3531 (50.7)	793 (47.4)
Race and ethnicity				
African American	9311 (13.6)	8156 (13.7)	960 (13.8)	195 (11.6)
Asian	3666 (5.4)	3476 (5.8)	135 (1.9)	54 (3.2)
Hispanic	16 369 (24.0)	14 260 (23.9)	1645 (23.6)	458 (27.4)
Multiple races	2266 (3.3)	1902 (3.2)	284 (4.1)	79 (4.7)
White	35 920 (52.6)	31 204 (52.3)	3854 (55.3)	863 (51.5)
Other^e^	730 (1.1)	614 (1.0)	93 (1.3)	27 (1.6)
Grade level				
≤5	225 (0.3)	221 (0.4)	1 (<0.1)	0
6-8	22 540 (33.0)	21 999 (36.9)	473 (6.8)	68 (4.1)
9-12	37 941 (55.6)	31 263 (52.4)	5433 (77.9)	1244 (74.3)
Other	7557 (11.1)	6129 (10.3)	1066 (15.3)	363 (21.7)
Community type				
Large metropolitan	38 432 (56.3)	33 511 (56.2)	3930 (56.4)	990 (59.1)
Small metropolitan	20 254 (29.7)	17 629 (29.6)	2121 (30.4)	507 (30.3)
Nonmetropolitan	9577 (14.0)	8478 (14.2)	921 (13.2)	178 (10.6)
Total family income, $				
<20 000	10 451 (15.3)	9092 (15.3)	1104 (15.8)	256 (15.3)
20 000-50 000	18 752 (27.5)	16 299 (27.3)	1951 (28.0)	500 (29.8)
>50 000-75 000	9803 (14.4)	8579 (14.4)	981 (14.1)	247 (14.8)
>75 000	29 258 (42.9)	25 647 (43.0)	2935 (42.1)	672 (40.1)
Age, mean (SD), y				
At NSDUH completion	14.5 (1.7)	14.4 (1.7)	15.8 (1.2)	15.8 (1.1)
At first cannabis use	NA	NA	14.2 (1.7)	13.4 (1.8)

Abbreviations: NA, not applicable; NSDUH, National Survey on Drug Use and Health.

^a^ From the composite of 2015 to 2019 National Survey on Drug Use and Health (NSDUH) data sets. Adjusted using individual respondent weights to represent the US national population; additional information is provided elsewhere.^21^

^b^ Nonuse is defined as denying any lifetime use of cannabis or not having used cannabis in the past 12 months.

^c^ Nondisordered cannabis use is defined as having used cannabis sometime in the past 12 months, without meeting criteria for cannabis use disorder.

^d^ Cannabis use disorder is defined as having met use disorder criteria established by the *Diagnostic and Statistical Manual of Mental Disorders (Fourth Edition, Text Revision)*.^13^

^e^ Other race and ethnicity category included American Indian or Alaska Native and Native Hawaiian or other Pacific Islander.

### Prevalence of Psychosocial Events

**Figure 1.**
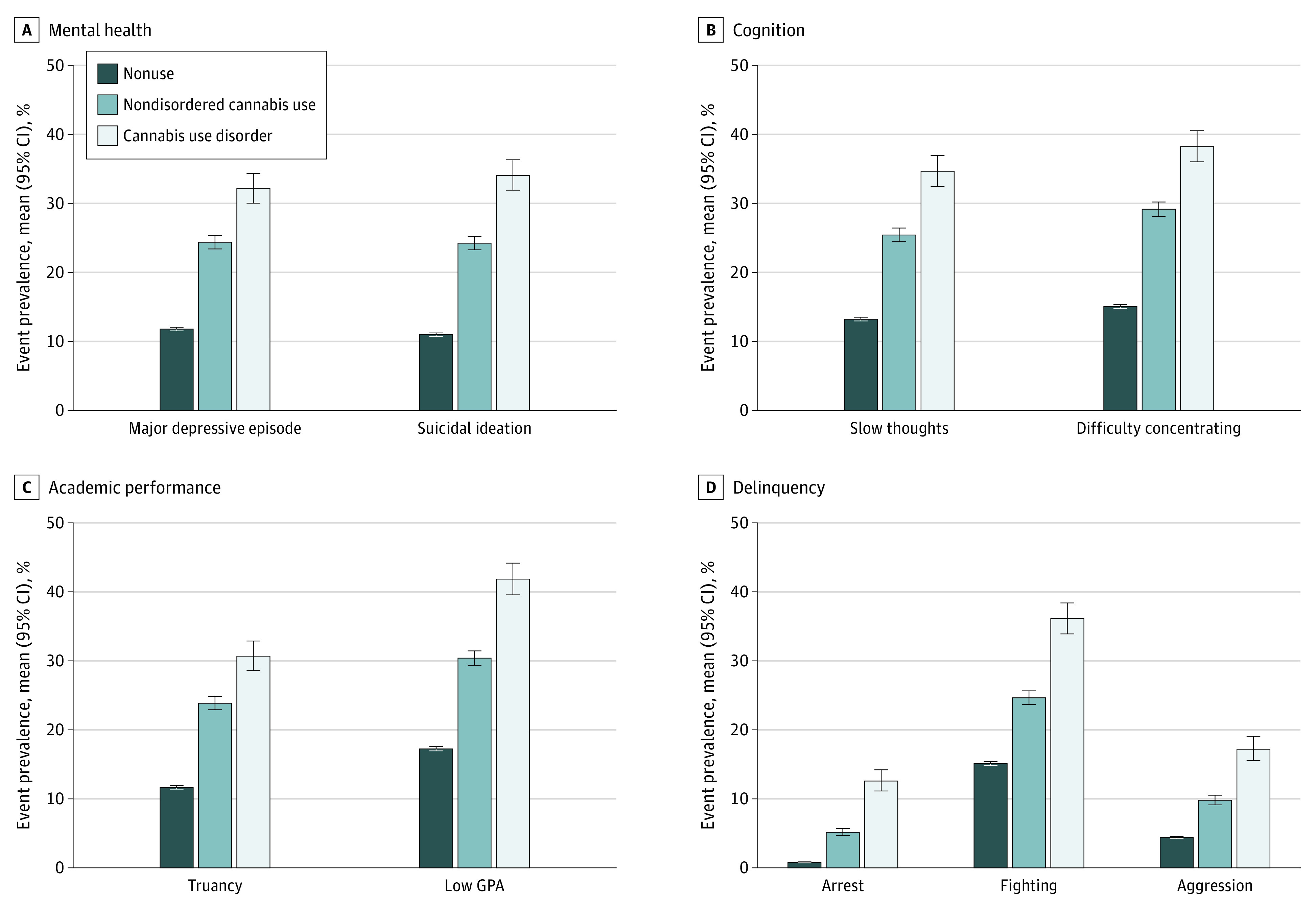
Prevalence of Adverse Adolescent Psychosocial Events Among Nonuse, Nondisordered Cannabis Use, and Cannabis Use Disorder Groups Data are from the 2015 to 2019 National Surveys on Drug Use and Health. GPA indicates grade point average.

Prevalence of adverse psychosocial events for the nonuse group ranged from 0.8% to 17.3%. For individuals with NDCU, prevalence ranged from 5.2% to 30.4%. For individuals with CUD, prevalence ranged from 12.6% to 41.9%. All NDCU and CUD measures were statistically larger than those in the nonuse group (Figure 1).

### Associations With Adverse Psychosocial Events

#### Poor Mental Health

Compared with nonuse, the unadjusted odds of a recent MDE were higher for both NDCU (OR, 2.40; 95% CI, 2.15-2.67) and CUD (OR, 3.48; 95% CI, 2.95-4.11) groups (Table 2). After adjusting for age, sex, race and ethnicity, and AUD, and compared with nonuse, the ND-CU group had nearly twice the odds of experiencing at least 1 MDE (aOR, 1.86; 95% CI, 1.67-2.08) (Table 3).

**Table 2.** Associations of Degrees of Adolescent Cannabis Use With Adolescent Psychosocial Events^a^

Adverse adolescent psychosocial event	Nondisordered cannabis use (n = 6971)^b^		Cannabis use disorder (n = 1675)^c^	
	OR (95% CI)	*P* value	OR (95% CI)	*P* value
Mental health				
≥1 MDE in the past year	2.40 (2.15-2.67)	<.001	3.48 (2.95-4.11)	<.001
Recent suicidal ideation	2.63 (2.39-2.90)	<.001	4.01 (3.42-4.70)	<.001
Cognition				
Recent slower thoughts	2.28 (2.05-2.53)	<.001	3.35 (2.87-3.92)	<.001
Recent difficulty concentrating	2.35 (2.13-2.59)	<.001	3.35 (2.89-3.89)	<.001
Academic performance				
≥1 d of school skipped in the past month	2.36 (2.08-2.69)	<.001	4.40 (3.72-5.20)	<.001
≤C+ GPA in the past semester	1.94 (1.75-2.15)	<.001	3.56 (3.12-4.06)	<.001
Delinquency				
≥1 arrests for breaking the law in the past year	5.60 (4.34-7.21)	<.001	17.72 (14.03-22.38)	<.001
≥1 serious fights in the past year	1.67 (1.49-1.88)	<.001	3.38 (2.92-3.93)	<.001
≥1 attacks with intent to harm in the past year	1.92 (1.61-2.28)	<.001	4.72 (3.92-5.69)	<.001

Abbreviations: GPA, grade point average; MDE, major depressive episode; OR, odds ratio.

^a^ From the composite of 2015 to 2019 National Survey on Drug Use and Health data sets. The comparison group was no cannabis use.

^b^ Nondisordered cannabis use is defined as having used cannabis sometime in the past 12 months, without meeting criteria for cannabis use disorder.

^c^ Cannabis use disorder is defined as having met use disorder criteria established by the *Diagnostic and Statistical Manual of Mental Disorders (Fourth Edition, Text Revision)*.^13^

**Table 3.** Adjusted Associations Between Degrees of Adolescent Cannabis Use and Adolescent Psychosocial Events^a^

Adverse adolescent psychosocial event	Nondisordered cannabis use (n = 6971)^b^		Cannabis use disorder (n = 1675)^c^	
	aOR (95% CI)	*P* value	aOR (95% CI)	*P* value
Mental health				
≥1 MDE in the past year	1.86 (1.67-2.08)	<.001	2.42 (2.02-2.89)	<.001
Recent suicidal ideation	2.08 (1.88-2.29)	<.001	2.92 (2.43-3.50)	<.001
Cognition				
Recent slower thoughts	1.76 (1.58-1.96)	<.001	2.37 (2.00-2.80)	<.001
Recent difficulty concentrating	1.81 (1.65-2.00)	<.001	2.37 (2.01-2.80)	<.001
Academic performance				
≥1 d of school skipped in the past month	1.90 (1.67-2.16)	<.001	2.95 (2.43-3.57)	<.001
≤C+ GPA in the past semester	1.80 (1.62-2.00)	<.001	3.11 (2.69-3.59)	<.001
Delinquency				
≥1 arrests for breaking the law in the past year	4.15 (3.17-5.43)	<.001	10.47 (7.81-14.03)	<.001
≥1 serious fights in the past year	2.04 (1.80-2.31)	<.001	3.61 (3.07-4.26)	<.001
≥1 attacks with intent to harm in the past year	2.16 (1.79-2.62)	<.001	4.33 (3.43-5.47)	<.001

Abbreviations: aOR, adjusted odds ratio; GPA, grade point average; MDE, major depressive episode.

^a^ From the composite of 2015 to 2019 National Survey on Drug Use and Health data sets. The comparison group was no cannabis use. aORs controlled for age, sex, race and ethnicity, and alcohol use disorder.

^b^ Nondisordered cannabis use is defined as having used cannabis sometime in the past 12 months, without meeting criteria for cannabis use disorder.

^c^ Cannabis use disorder is defined as having met use disorder criteria established by the *Diagnostic and Statistical Manual of Mental Disorders (Fourth Edition, Text Revision)*.^13^

Odds of recent SI were higher in the NDCU (2.63; 95% CI, 2.39-2.90) and CUD (OR, 4.01; 95% CI, 3.42-4.70) groups compared with the nonuse group (Table 2). After adjusting, the NDCU group had approximately twice the odds of SI (aOR, 2.08; 95% CI, 1.88-2.29) (Table 3).

#### Cognitive Deficits

Odds of endorsing slowed thinking were greater for NDCU (OR, 2.28; 95% CI, 2.05-2.53) and CUD (OR, 3.35; 95% CI, 2.87-3.92) groups than for the nonuse group (Table 2). After adjusting, odds of slowed thinking remained higher for adolescents with NDCU (aOR, 1.76; 95% CI, 1.58-1.96) and CUD (aOR, 2.37; 95% CI, 2.00-2.80) (Table 3). Odds of difficulty concentrating were significantly elevated for both NDCU (OR, 2.35; 95% CI, 2.13-2.59) and CUD (OR, 3.35; 95% CI, 2.89-3.89) groups (Table 2). After adjusting for the potentially confounding effects of demographic factors and AUD, the NDCU group had nearly 2 times the odds of difficulty concentrating than nonusers (aOR, 1.81; 95% CI, 1.65-2.00) (Table 3).

#### Low Academic Performance

Odds of truancy were significantly higher in the NDCU (OR, 2.36; 95%CI, 2.08-2.69) and CUD (OR, 4.40; 95% CI, 3.72-5.20) groups compared with nonuse. After adjusting for demographic factors and AUD and compared with nonuse, NDCU was associated with nearly twice the odds of truancy (aOR, 1.90; 95% CI, 1.67-2.16) (Table 3).

Odds of low academic performance for NDCU and CUD groups were significantly greater than controls (Table 2). Adjusting for demographic factors and AUD lowered these odds for the NDCU (aOR, 1.80; 95% CI, 95% CI, 1.62-2.00) and CUD (aOR, 3.11; 95% CI, 2.69-3.59) groups, but risk remained statistically significant. CUD had a greater strength of association with low academic performance than NDCU did (Table 2 and Table 3).

#### Delinquent Behavior

Regarding past-year arrests for breaking the law, risks were significantly higher for the NDCU (OR, 5.60; 95% CI, 4.34-7.21) and CUD (OR, 17.72; 95% CI, 14.03-22.38) groups (Table 2). After adjusting for potential demographic confounders and AUD, odds of arrest were reduced but remained statistically significant in both NDCU (aOR, 4.15; 95% CI, 3.17-5.43) and CUD (aOR, 10.47; 95% CI, 7.81-14.03) groups (Table 3). The association of cannabis use with risk of arrest was stronger for the CUD group than for the NDCU group (Table 2 and Table 3).

Adolescents with NDCU or CUD, compared with those without cannabis use, had significantly greater odds of any serious fighting in the past year (Table 2 and Table 3). NDCU and CUD groups were significantly different for past-year serious fighting (Table 3).

When examining NDCU and CUD use regarding odds of physical aggression, odds were greater than those for nonusers (NDCU: aOR, 2.16; 95% CI, 1.79-2.62; CUD: aOR, 4.33; 95% CI, 3.43-5.47). The association of cannabis use with physical aggression was stronger for CUD than for NDCU (Table 2 and Table 3).

#### Stratification by Sex, Age, and Cannabis Degree of Use

eTable 3 in Supplement 1 stratifies NDCU and CUD by sex. Female respondents had higher risk of MDE and SI than male respondents in both NDCU and CUD groups. eTable 4 in Supplement 1 stratifies NDCU and CUD by age groups of 12 to 14 years and 15 to 17 years; no significant differences between age groups were indicated. eTable 5 in Supplement 1 further stratifies adolescents into exclusive cannabis use groups by recency of cannabis use (ie, lifetime, past-year, or past-month). Interestingly, past-year CUD and NDCU were associated with higher risk of MDE and SI compared with past-month CUD and NDCU (eTable 5 in Supplement 1).

### Cannabis Use Frequency by Degree of Adolescent Cannabis Use

Frequency of cannabis use, defined as number of days used per time frame (week, month, year), was consistently 3 times higher for CUD than NDCU across all time frames (eTable 6 in Supplement 1). Yearly mean use was 152.5 (95% CI, 146.8-158.3) days for CUD and 58.8 (95% CI, 56.6-61.0) days for NDCU (eTable 6 in Supplement 1).

### Substance Use Disorder Criteria

**Figure 2.**
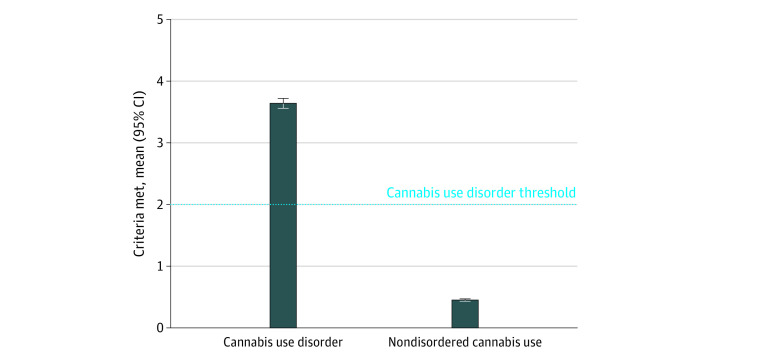
Mean Number of *Diagnostic and Statistical Manual of Mental Disorders (Fourth Edition, Text Revision)*^13^ Cannabis Use Disorder Criteria Met by Degree of Adolescent Cannabis Use

Adolescents with CUD met a mean of 3.64 (95% CI, 3.56-3.72) *DSM-5* criteria.^13^ Adolescents with NDCU met a mean of 0.45 (95% CI, 0.43-0.47) criteria (Figure 2).

## Discussion

This cross-sectional study within a large, nationally representative sample of US adolescents found that NDCU was approximately 4 times more common than CUD. Adolescent NDCU was significantly associated with all adverse psychosocial correlates examined. For all adverse events (except MDE), the magnitudes of the associations of adolescent NDCU and CUD with adverse psychosocial events were significantly different. Finally, despite having increased odds of adverse psychosocial events, most adolescents with NDCU endorsed approximately one-fourth of the minimum *DSM-5* criteria for CUD.

Cannabis use among adolescents was associated with greater odds of MDE and SI. These findings are in line with past studies examining depression and suicidality associated with cannabis use.^9,14^ Presence of markers associated with poor adolescent mental health, such as depression and suicidality, are associated with meaningful long-term adverse educational and occupational attainment outcomes and increased risk of harmful substance use in adulthood.^25,26^ Strikingly, adolescents with NDCU and CUD did not significantly differ on measures of MDE.^27^

Elevated rates of MDE and SI among both cannabis use populations could be due to unexamined factors, such as anxiety. Cannabis use in adolescence may represent self-treatment to ameliorate mood symptoms and is also associated with developing major depression.^28^ Recent studies have suggested that while cannabis use may ameliorate mood symptoms, ongoing use worsens mood symptoms.^29,30^ Building on past studies,^9,14,31^ our findings support NDCU as a clinically relevant risk marker associated with major depression and suicidality. Given public perspectives on cannabis as a treatment for depressive symptoms, future longitudinal research is necessary to better describe this association.

Adolescent cannabis use was associated with worse executive control, decreased attention, and deficits in episodic memory.^16,32^ Compared with nonuse, adolescents in ND-CU and CUD groups were approximately 2 to 3 times more likely to experience cognitive deficits. A potential confounder of these associations is that the cognitive effects measured in this study are also markers associated with other mental health conditions, such as attention deficit/hyperactivity disorder, which is itself a risk factor associated with cannabis use.^33^

Irrespective of potential confounding, identifying NDCU in community practice may be a risk marker associated with impairments in cognition. Past studies in young adult and adult populations have highlighted the associations of cannabis use with cognitive deficits, even beyond 30 days of abstinence.^34,35^ Consistent with that finding is that chronic cannabis use is associated with decreased hippocampal volume by midlife.^36,37^ Future studies could examine how length of cannabis abstinence interacts with cognition across varying age groups.

Truancy during adolescence has long-lasting associations with adverse life outcomes, including crime and problematic substance use.^38^ In our study, adolescent cannabis users were between 2.5 and 4.5 times more likely to be truant from school than were their nonuser peers.

Numerous studies have reported that adolescent cannabis use is linked to poor academic performance.^15,17,22^ In this study, adolescent cannabis users were 2 to 3.5 times more likely to report a low GPA than their nonuser peers, which is similar to past longitudinal studies of high school students.^15^ Initiating measures that strictly limited access to cannabis for adolescents has been shown to be associated with improved academic performance.^39^

Demonstrating a causal link between adolescent cannabis exposure in both the CUD and NDCU groups and adverse psychosocial outcomes is not possible with the current study. Nevertheless, lines of research in neuroscience demonstrate that recreational cannabis use in adolescents is associated with decreased brain volumes in CB1-rich areas of the brain involved in motivational, emotional, and affective processing.^40^ Furthermore, earlier age of onset of cannabis use also is associated with the magnitude of these changes. Prospective longitudinal research with repeated measures could help distinguish explanations for the fairly strong associations observed in this study.

Subdiagnostic cannabis use (ie, NDCU) was associated with significantly increased risks of all adverse psychosocial events, compared with nonuse, making NDCU a risk marker associated with adverse psychosocial events in adolescence. We observed a stepwise severity gradient for the odds of psychosocial associations among nonuse, NDCU, and CUD. This severity gradient was also observed in prevalence values for adverse psychosocial events across all degrees of cannabis use. Furthermore, this observation was corroborated by a stepwise cannabis use frequency trend between NDCU and CUD.

Substance use disorders, including cannabis, are underdiagnosed,^41^ particularly in adolescents. Our repeated finding that NDCU was significantly associated with psychosocial risks compared with nonuse underscores the vulnerability of adolescents with NDCU. First, if we assume that adolescents with NDCU, which is more common than CUD among adolescents, have sufficient adverse effects associated with their cannabis use to meet criteria for pathological use, then many US adolescents are not being identified in community practice. In fact, according to our data, the US adolescent population includes 2.5 million adolescents with NDCU.

The observed poor differentiation between NDCU and CUD among adolescents could be the result of inaccurate reporting by adolescents. Interestingly, individuals with CUD met a mean of 3.6 *DSM-5*^13^ CUD criteria, while individuals with NDCU only met a mean of 0.5 criteria. A 2002 study by Degenhardt et al^42^ found that strict adherence to *Diagnostic and Statistical Manual of Mental Disorders* (*Fourth Edition*) criteria was associated with underdiagnosis of cannabis use in adolescents.^42^ It is also possible that the current diagnostic criteria for evaluating adolescents’ cannabis use are not developmentally sensitive.

### Limitations

This study’s findings should be interpreted in the context of several important limitations. First, the study was based on self-report, which may not correspond with objective clinical assessments. As a result, this study may underreport cannabis use, as individuals regularly underreport their substance use.^43^ Future studies may consider a combination of self-report and drug screens.^44^ Second, this is a cross-sectional study; therefore, we are limited in our ability to assess the direction of causality of reported associations. Particularly, there remains the possibility that adolescent cannabis use and adverse events have similar causes (eg, stress-related cannabis use^45^). Third, given limitations in NSDUH data, we are unable to adjust for psychiatric comorbidities, such as anxiety, and prior research suggests that anxiety predates substance use.^46,47^ If anxiety were a common cause of both cannabis use and psychosocial events, it might partially or even fully account for the observed associations between cannabis use and psychosocial events. Fourth, lack of statistical differences between certain groups may be a function of low frequency of adverse events affecting power. Fifth, this study does not differentiate methods of cannabis use (eg, combusted, edible, or vaporized).

## Conclusions

In this cross-sectional study of US adolescents, individuals with subthreshold NDCU, compared with nonusers, had greater odds of adverse psychosocial events, and for several adverse events, the risk among individuals with NDCU did not significantly differ from that among individuals with CUD. Strong associations between NDCU and adverse psychosocial events have implications in clinical practice. These findings support NDCU as a useful clinical risk marker associated with a range of adverse psychosocial events. With growing US acceptance of cannabis in both medicinal and recreational settings, clinicians should be vigilant to screen, evaluate, and treat cannabis use in adolescents.

## Notes

Supplement 1. **eTable 1.** National Survey on Drug Use and Health Variables for Classification of Nonuse, Nondisordered Cannabis Use, and Cannabis Use Disorder **eTable 2.** National Survey on Drug Use and Health (NSDUH) Variables for Adverse Adolescent Events **eTable 3.** Associations of Degrees of Adolescent Cannabis Use With Adolescent Psychosocial Events Stratified by Sex **eTable 4.** Associations of Degrees of Adolescent Cannabis Use With Adolescent Psychosocial Events Stratified by Age **eTable 5.** Associations of Recency and Degree of Adolescent Cannabis Use With Psychosocial Events **eTable 6.** Cannabis Use Frequency by Degree of Adolescent Cannabis Use

Supplement 2.
Data Sharing Statement
