# Supplementary material for: Nondisordered Cannabis Use Among US Adolescents
Source: JAMA Netw Open. 2023 May 3;6(5):e2311294. doi: 10.1001/jamanetworkopen.2023.11294 (PMC10157425; doi:10.1001/jamanetworkopen.2023.11294)
Supplement: Supplement 1. — eTable 1. National Survey on Drug Use and Health Variables for Classification of Nonuse, Nondisordered Cannabis Use, and Cannabis Use Disorder eTable 2. National Survey on Drug Use and Health (NSDUH) Variables for Adverse Adolescent Events eTable 3. Associations of Degrees of Adolescent Cannabis Use With Adolescent Psychosocial Events Stratified by Sex eTable 4. Associations of Degrees of Adolescent Cannabis Use With Adolescent Psychosocial Events Stratified by Age eTable 5. Associations of Recency and Degree of Adolescent Cannabis Use With Psychosocial Events eTable 6. Cannabis Use Frequency by Degree of Adolescent Cannabis Use [file jamanetwopen-e2311294-s001.pdf]

Supplemental Online Content

Sultan RS, Zhang AW, Olfson M, Kwizera MH, Levin FR. Nondisordered cannabis use among US adolescents. *JAMA Netw Open*. 2023;6(5):e2311294.
doi:10.1001/jamanetworkopen.2023.11294

eTable 1. National Survey on Drug Use and Health Variables for Classification of Nonuse, Nondisordered Cannabis Use, and Cannabis Use Disorder
eTable 2. National Survey on Drug Use and Health (NSDUH) Variables for Adverse Adolescent Events
eTable 3. Associations of Degrees of Adolescent Cannabis Use With Adolescent Psychosocial Events Stratified by Sex
eTable 4. Associations of Degrees of Adolescent Cannabis Use With Adolescent Psychosocial Events Stratified by Age
eTable 5. Associations of Recency and Degree of Adolescent Cannabis Use With Psychosocial Events
eTable 6. Cannabis Use Frequency by Degree of Adolescent Cannabis Use

This supplemental material has been provided by the authors to give readers additional information about their work.

© 2023 Sultan RS et al. *JAMA Network Open.*

eTable 1. National Survey on Drug Use and Health Variables for Classification of Nonuse, Nondisordered Cannabis Use, and Cannabis Use Disorder

<table>
<tr><th>Degree of cannabis use</th><th>Codebook variable</th><th>Description</th></tr>
<tr><td>Non-use</td><td>(irmjrc == 9 & abodmrj == 0) |
(irmjrc == 3 & abodmrj == 0)</td><td>No lifetime cannabis use OR non-recent use (>365 days ago)</td></tr>
<tr><td>Non-Disordered Cannabis Use (ND-CU)</td><td>(irmjrc == 2 & abodmrj == 0)</td><td>Cannabis used “more than 30 days ago but within the past 12 mos”, AND absence of CUD (based on DSM-IV criteria)</td></tr>
<tr><td>Cannabis Use Disorder (CUD)</td><td>abodmrj == 1</td><td>Has CUD (based on DSM-IV criteria)</td></tr>
</table>

© 2023 Sultan RS et al. *JAMA Network Open.*

eTable 2. National Survey on Drug Use and Health (NSDUH) Variables for Adverse Adolescent Events

Adverse adolescent event	Codebook variable	Description
One or more major depressive episodes (MDE) in the past year	ymdeyr == 1	Past year Major Depressive Episode (MDE)
Recent suicidal ideation	yowrdbtr == 1	During that period, did you ever think that it would be better if you were dead?
Recent slower thoughts	yowrthot == 1	On most days during that [TIMEFILL] time, did your thinking seem slower than usual or seem mixed up?
Recent difficulty concentrating	yowrconc == 1	On most days, did you have a lot more trouble than usual keeping your mind on things?
One or more days of school skipped in the past month (as a full time student)	eduskpmon >= 1 & eduskpmon <= 30 & edufulpar == 1	During the past 30 days, that is since [DATEFILL], how many whole days did you miss because you skipped or "cut" or just didn't there? Please do not include days you missed because you stayed home with a sick child or other family member.
C+ grade average or below in the past semester	yelstgrd == 3 | yelstgrd == 4	What were your grades for the last semester or grading period you completed?
One or more arrests for breaking the law in the past year	NOBOOKY2 >= 1 & NOBOOKY2 <= 3	Not counting minor traffic violations, how many times during the past 12 months have you been arrested and booked for breaking a law?
One or more serious fights in the past year	yeyfgtsw >= 2 & yeyfgtsw <= 5	During the past 12 months, how many times have you gotten into a serious fight at school or work?
One or more attacks with intent to harm in the past year	yeyattak > 1	During the past 12 months, how many times have you attacked someone with the intent to seriously hurt them?

© 2023 Sultan RS et al. *JAMA Network Open.*

eTable 3. Associations of Degrees of Adolescent Cannabis Use With Adolescent Psychosocial Events[a,b,c] Stratified by Sex

	**Non-Disordered Cannabis Use[d]**						**Cannabis Use Disorder[e]**					
	**Female (N = 3,531)**			**Male (N=3,440)**			**Female (N=793)**			**Male (N=882)**		
**Adverse Adolescent Psychosocial Event**	**aOR**	**95% CI**	**p**	**aOR**	**95% CI**	**p**	**aOR**	**95% CI**	**p**	**aOR**	**95% CI**	**p**
Mental Health												
One or more major depressive episodes (MDE) in the past year	2.06	[1.80, 2.36]	<0.001	1.47	[1.15, 1.86]	<0.001	2.56	[2.09, 3.13]	<0.001	2.15	[1.65, 2.80]	<0.001
Recent suicidal ideation	2.35	[2.05, 2.68]	<0.001	1.51	[1.21, 1.88]	<0.001	3.31	[2.67, 4.11]	<0.001	2.29	[1.80, 2.91]	<0.001
Cognition												
Recent slower thoughts	2.01	[1.76, 2.29]	<0.001	1.32	[1.05, 1.65]	<0.001	2.69	[2.20, 3.30]	<0.001	1.91	[1.51, 2.42]	<0.001
Recent difficulty concentrating	2.11	[1.86, 2.39]	<0.001	1.33	[1.09, 1.62]	<0.001	2.67	[2.14, 3.33]	<0.001	1.98	[1.65, 2.38]	<0.001
Academic Performance												
One or more days of school skipped in the past month	2.16	[1.87, 2.51]	<0.001	1.59	[1.31, 1.93]	<0.001	2.80	[2.20, 3.57]	<0.001	3.12	[2.41, 4.03]	<0.001
C+ grade average or below in the past semester	1.89	[1.61, 2.20]	<0.001	1.73	[1.52, 1.98]	<0.001	3.39	[2.63, 4.37]	<0.001	2.89	[2.41, 3.46]	<0.001
Delinquency												
One or more arrests for breaking the law in the past year	3.57	[2.11, 6.04]	<0.001	4.50	[3.31, 6.11]	<0.001	10.85	[6.25, 18.82]	<0.001	10.32	[7.35, 14.50]	<0.001
One or more serious fights in the past year	2.45	[2.02, 2.97]	<0.001	1.71	[1.46, 1.99]	<0.001	4.05	[3.16, 5.19]	<0.001	3.32	[2.69, 4.10]	<0.001
One or more attacks with intent to harm in the past year	2.64	[1.99, 3.49]	<0.001	1.86	[1.46, 2.37]	<0.001	4.72	[3.28, 6.79]	<0.001	4.09	[3.01, 5.56]	<0.001

[a]From the composite of 2015-2019 National Survey on Drug Use and Health (NSDUH) datasets
[b]The comparison group is non-use
[c]Controlled for age, sex, race/ethnicity, and alcohol use disorder to obtain adjusted odds ratios (aOR)
[d]Non-Disordered Cannabis Use is defined as having used cannabis sometime in the past 12 months, without meeting criteria for Cannabis Use Disorder
[e]Cannabis Use Disorder is defined as having met use disorder criteria established by the DSM-IV (American Psychological Association, 1994)

© 2023 Sultan RS et al. *JAMA Network Open.*

eTable 4. Associations of Degrees of Adolescent Cannabis Use With Adolescent Psychosocial Events[a,b,c] Stratified by Age

	Non-Disordered Cannabis Use[d]						Cannabis Use Disorder[e]					
	Age 12-14 (n=1201)			Age 15-17 (n=6162)			Age 12-14 (n=216)			Age 15-17 (n=1557)		
**Adverse Adolescent Psychosocial Event**	**aOR**	**95% CI**	**p**	**aOR**	**95% CI**	**p**	**aOR**	**95% CI**	**p**	**aOR**	**95% CI**	**p**
Mental Health												
One or more major depressive episodes (MDE) in the past year	2.34	[2.05, 2.68]	<0.001	2.19	[1.89, 2.55]	<0.001	2.91	[1.95, 4.34]	<0.001	2.77	[1.86, 4.14]	<0.001
Recent suicidal ideation	2.36	[2.01, 2.77]	<0.001	2.30	[1.93, 2.74]	<0.001	2.81	[1.74, 4.55]	<0.001	2.75	[1.72, 4.41]	<0.001
Cognition												
Recent slower thoughts	1.98	[1.70, 2.30]	<0.001	1.95	[1.63, 2.32]	<0.001	2.52	[1.81, 3.49]	<0.001	2.61	[1.86, 3.67]	<0.001
Recent difficulty concentrating	2.19	[1.93, 2.50]	<0.001	2.15	[1.86, 2.50]	<0.001	2.76	[2.04, 3.74]	<0.001	2.78	[1.98, 3.90]	<0.001
Academic Performance												
One or more days of school skipped in the past month	1.84	[1.49, 2.27]	<0.001	1.71	[1.37, 2.14]	<0.001	3.17	[2.28, 4.41]	<0.001	2.67	[1.84, 3.86]	<0.001
C+ grade average or below in the past semester	1.84	[1.55, 2.17]	<0.001	1.70	[1.40, 2.07]	<0.001	3.35	[2.59, 4.35]	<0.001	3.15	[2.32, 4.27]	<0.001
Delinquency												
One or more arrests for breaking the law in the past year	3.18	[1.99, 5.08]	<0.001	2.74	[1.70, 4.40]	<0.001	10.58	[4.71, 23.79]	<0.001	8.55	[3.32, 22.01]	<0.001
One or more serious fights in the past year	2.40	[1.99, 2.89]	<0.001	2.31	[1.81, 2.93]	<0.001	3.80	[3.02, 4.77]	<0.001	3.80	[2.80, 5.17]	<0.001
One or more attacks with intent to harm in the past year	2.08	[1.62, 2.67]	<0.001	2.09	[1.43, 3.06]	<0.001	3.77	[2.63, 5.42]	<0.001	3.58	[2.31, 5.57]	<0.001

[a]From the composite of 2015-2019 National Survey on Drug Use and Health (NSDUH) datasets
[b]The comparison group is non-use
[c]Controlled for age, sex, race/ethnicity, and alcohol use disorder to obtain adjusted odds ratios (aOR)
[d]Non-Disordered Cannabis Use is defined as having used cannabis sometime in the past 12 months, without meeting criteria for Cannabis Use Disorder
[e]Cannabis Use Disorder is defined as having met use disorder criteria established by the DSM-IV (American Psychological Association, 1994)

© 2023 Sultan RS et al. *JAMA Network Open.*

eTable 5. Associations of Recency and Degree of Adolescent Cannabis Use With Psychosocial Events[a,b,c]

	**Non-Recent Use (N = 2081)**			**Non-Disordered Cannabis Use Past Year (N = 3736)**			**Non-Disordered Cannabis Use Past Month (N = 3627)**			**Cannabis Use Disorder Past Year (N = 394)**			**Cannabis Use Disorder Past Month (N = 1379)**		
**Adverse Adolescent Psychosocial Event**	**aOR**	**95% CI**	**p**	**aOR**	**95% CI**	**p**	**aOR**	**95% CI**	**p**	**aOR**	**95% CI**	**p**	**aOR**	**95% CI**	**p**
Mental Health															
One or more major depressive episodes (MDE) in the past year	2.21	[1.90, 2.58]	<0.001	2.00	[1.79, 2.23]	<0.001	2.02	[1.77, 2.31]	<0.001	3.28	[2.53, 4.27]	<0.001	2.54	[2.09, 3.09]	<0.001
Recent suicidal ideation	2.07	[1.76, 2.44]	<0.001	2.21	[2.00, 2.44]	<0.001	2.24	[1.96, 2.56]	<0.001	4.12	[3.04, 5.59]	<0.001	2.90	[2.38, 3.55]	<0.001
Cognition															
Recent slower thoughts	1.97	[1.72, 2.25]	<0.001	1.85	[1.67, 2.06]	<0.001	1.78	[1.59, 1.99]	<0.001	2.95	[2.10, 4.14]	<0.001	2.41	[2.03, 2.88]	<0.001
Recent difficulty concentrating	2.10	[1.81, 2.43]	<0.001	1.92	[1.74, 2.11]	<0.001	1.95	[1.75, 2.18]	<0.001	3.06	[2.20, 4.24]	<0.001	2.43	[2.06, 2.86]	<0.001
Academic Performance															
One or more days of school skipped in the past month	2.08	[1.80, 2.40]	<0.001	2.01	[1.76, 2.29]	<0.001	2.60	[2.27, 2.97]	<0.001	2.72	[1.90, 3.89]	<0.001	3.35	[2.75, 4.08]	<0.001
C+ grade average or below in the past semester	2.48	[2.23, 2.76]	<0.001	1.93	[1.73, 2.15]	<0.001	2.15	[1.94, 2.38]	<0.001	3.19	[2.44, 4.17]	<0.001	3.44	[2.93, 4.04]	<0.001
Delinquency															
One or more arrests for breaking the law in the past year	7.92	[5.51, 11.37]	<0.001	5.99	[4.49, 7.98]	<0.001	9.55	[7.31, 12.47]	<0.001	20.92	[13.18, 33.21]	<0.001	14.22	[10.11, 20.00]	<0.001
One or more serious fights in the past year	2.49	[2.23, 2.77]	<0.001	2.19	[1.93, 2.48]	<0.001	2.87	[2.57, 3.19]	<0.001	3.44	[2.59, 4.57]	<0.001	4.08	[3.38, 4.92]	<0.001
One or more attacks with intent to harm in the past year	3.24	[2.62, 4.01]	<0.001	2.41	[2.00, 2.92]	<0.001	3.66	[3.02, 4.42]	<0.001	4.40	[2.84, 6.80]	<0.001	5.09	[4.02, 6.44]	<0.001

[a]From the composite of 2015-2019 National Survey on Drug Use and Health (NSDUH) datasets
[b]The comparison group is non-use
[c]Controlled for age, sex, race/ethnicity, and alcohol use disorder to obtain adjusted odds ratios (aOR)
[d]Non-Recent Use is defined as lifetime cannabis use, but not within the past 12 months

© 2023 Sultan RS et al. *JAMA Network Open.*

[e]Non-Disordered Cannabis Use Past Year is defined as having used cannabis within the past 12 months, not within the past 30 days, and without Cannabis Use Disorder
[f]Non-Disordered Cannabis Use Past Month is defined as having used cannabis within the past 30 days, without Cannabis Use Disorder
[g]Cannabis Use Disorder Past Year is defined as having used cannabis within the past 12 months, not within the past 30 days, and with Cannabis Use Disorder
[h]Cannabis Use Disorder Past Month is defined as having used cannabis within the past 30 days, with Cannabis Use Disorder

© 2023 Sultan RS et al. *JAMA Network Open.*

eTable 6. Cannabis Use Frequency by Degree of Adolescent Cannabis Use

Cannabis Use Frequency	Non-Disordered Cannabis Use		Cannabis Use Disorder	
	**Mean**	**95% CI**	**Mean**	**95% CI**
Days per year	58.8	[56.6, 61.0]	152.5	[146.8, 158.3]
Days per month	3.9	[3.7, 4.0]	10.9	[10.4, 11.4]
Days per week	0.8	[0.8, 0.9]	2.2	[2.1, 2.3]

© 2023 Sultan RS et al. *JAMA Network Open.*
