# Supplementary material for: Nondisordered Cannabis Use Among US Adolescents
Source: JAMA Netw Open. 2023 May 3;6(5):e2311294. doi: 10.1001/jamanetworkopen.2023.11294 (PMC10157425; doi:10.1001/jamanetworkopen.2023.11294)
Supplement: Supplement 2. — Data Sharing Statement [file jamanetwopen-e2311294-s002.pdf]

Data Sharing Statement

Sultan. Nondisordered Cannabis Use Among US Adolescents. *JAMA Netw Open*. Published May 03, 2023. doi:10.1001/jamanetworkopen.2023.11294

Data
Data available: No

Additional Information
Explanation for why data not available: Data is already public (National Survey on Drug Use and Health; SAMHSA)
